# Bioactive Constituents from the Aerial Parts of *Lippia triphylla*

**DOI:** 10.3390/molecules201219814

**Published:** 2015-12-08

**Authors:** Yi Zhang, Yue Chen, Shiyu Wang, Yongzhe Dong, Tingting Wang, Lu Qu, Nan Li, Tao Wang

**Affiliations:** 1 Tianjin State Key Laboratory of Modern Chinese Medicine, 312 Anshanxi Road, Nankai District, Tianjin 300193, China; zhwwxzh@263.net (Y.Z.); chenyue19890126@sina.com (Y.C.); dongyongzhe44@hotmail.com (Y.D.); qululuhan88@163.com (L.Q.); 2 Tianjin Key Laboratory of TCM Chemistry and Analysis, Institute of Traditional Chinese Medicine, Tianjin University of Traditional Chinese Medicine, 312 Anshan Road, Nankai District, Tianjin 300193, China; 13622103155@163.com (S.W.); 18202682964@163.com (T.T.W.); linan20080402@163.com (N.L.)

**Keywords:** *Lippia triphylla*, lippianoside, structure elucidation, antioxidant, triglyceride accumulation inhibition, L6 cells, HepG2 cells

## Abstract

Five new compounds, lippianosides A (**1**), B (**2**), C (**3**), D (**4**), and E (**5**), along with 26 (**6**–**31**) known ones were obtained from the 95% EtOH extract of *Lippia triphylla* (*L. triphylla*) aerial parts collected from Rwanda, Africa. Among the known compounds, **11** and **17**–**30** were isolated from the *Lippia* genus for the first time. In addition, **12**, **13**, and **16** were firstly obtained from this species. The structures of them were elucidated by chemical and spectroscopic methods. The antioxidant and triglyceride accumulation inhibition effects of the 31 compounds were examined in L6 cells and HepG2 cells, respectively.

## 1. Introduction

The genus *Lippia* (*L.*) has a great economic value as a condiment and traditional medicine. *Lippia* leaves, flowers, and aerial parts were used in folk medicine for the treatment of respiratory and digestive system diseases [1]. Polyphenols from *L. citriodora* decreased triglyceride (TG) accumulation, the generation of reactive oxygen species (ROS) and restored mitochondrial membrane potential in adipocytes via ROS-mediated down-regulation of nuclear factor κB transcription factor, peroxisome proliferator-activated receptor γ-dependent transcription, upregulation of adiponectin and activation of AMP-activated protein kinase (AMPK) [2]. Essential oils from *L. thymoides* leaves had antimicrobial selectivity to Gram-positive bacteria *Staphylococcus aureus* and *Micrococcus luteus* [3]. Methanolic extract of *L. nodiflora* leaves showed reduced effect on ROS production against LPS induced toxicity in HepG2 cells [4]. *L. sidoides* displayed immunomodulatory effects through the inhibition of cyclic nucleotide-dependent phosphodiesterase activity and activation of p38 MAPK pathway [5]. *L. graveolens* extract and its constituents, cirsimaritin, hispidulin, and naringenin, could inhibit dipeptidyl peptidase IV and protein tyrosine phosphatase, which indicated that *L. graveolens* was useful for type 2 diabetes management [6]. Oral administration of γ-sitosterol isolated from *L. nodiflora* once daily for 21 days in STZ-induced diabetic rats resulted in a significant decrease in blood glucose and glycosylated hemoglobin with a significant increase in plasma insulin level, and, subsequently, increased insulin secretion in response to glucose [7].

*L. triphylla* (L′Her.) O. Kuntze (syn. *L. citrodora* (Ort.) HBK) is a perennial, bushy plant of *Verbenaceae* family, commonly named lemon verbena. It grows spontaneously in many countries in South America, such as Brazil, Chile, Argentina, and Peru, had been introduced into Europe by the end of the 17th century, and has since been cultivated in North Africa and Southern Europe [8]. It contains special lemon-like fragrance, and is used against vertigo, nausea, and headaches in Greece [9].

During the course of our studies, we identified five new compounds, lippianosides A–E (**1**–**5**), along with 26 known ones (**6**–**31**) from the 95% EtOH extract of *L. triphylla* aerial parts collected from Rwanda. Their structures were elucidated by chemical and spectroscopic methods. Based on previous *Lippia* genus activity reports evidence, the antioxidant and TG accumulation inhibitory effects of the isolates were examined.

## 2. Results and Discussion

**Figure 1:**
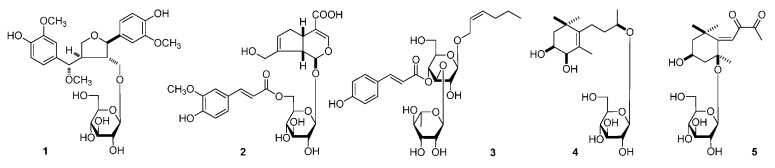
The new compounds (**1**–**5**) obtained from *L. triphylla.*

**Figure 2:**
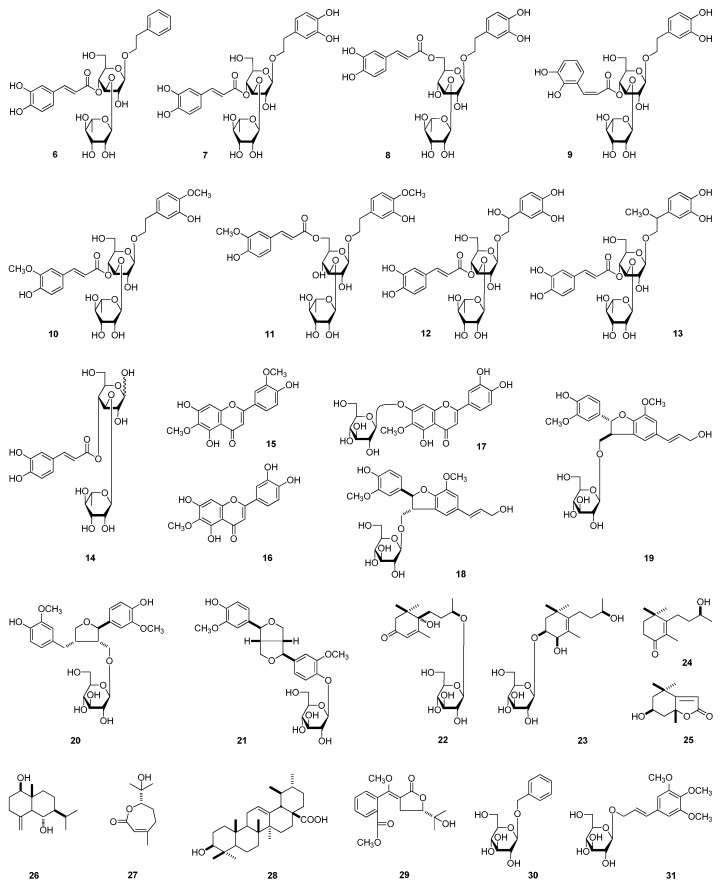
The known compounds (**6**–**31**) obtained from *L. triphylla*.

The 95% EtOH extract of *L. triphylla* was subjected to solvent partition, chromatographic isolation, and chemical and spectral analyses. As a result, five new compounds, lippianosides A–E (**1**–**5**) (Figure 1), together with 26 known ones (Figure 2), jionoside C(**6**) [10], *trans*-acteoside (**7**) [11], isoverbascoside (**8**) [12], *cis*-acteoside (**9**) [13], martynoside (**10**) [14], isomartynoside (**11**) [14], β-hydroxyacteoside (**12**) [15], campneoside I (**13**) [16], cistanoside F (**14**) [17], jaceosidin (**15**) [18], nepetin (**16**) [19], nepitrin (**17**) [20], dehydrodiconiferyl glucoside D (**18**) [21], dehydrodiconiferyl glucoside E (**19**) [21], (+)-lariciresinol-9-*O*-β-d-glucopyranoside (**20**) [22], (+)-pinoresinol 4-*O*-β-d-glucoside (**21**) [23,24], dihydrovomifoliol-*O*-β-d-glucopyranoside (**22**) [25,26], turpinionoside D (**23**) [27], 9-hydroxymegastigm-5-en-4-one (**24**) [28], (−)-loliolide (**25**) [29,30], eudesm-4(15)-ene-1β,6α-diol (**26**) [31], (6*S*)-3,7-dimethyl-7-hydroxy-2(*Z*)-octen-6-olide (**27**) [32], ursolic acid (**28**) [33], avicennone A (**29**) [34], benzyl alcohol *O*-β-d-glucopyranoside (**30**) [35], and icariside H_1_ (**31**) [36] were yielded and identified. Among the known ones, **11** and **17**–**30** were isolated from the *Lippia* genus for the first time, and **12**, **13**, and **16** were obtained from this species for the first time.

**Figure 3:**
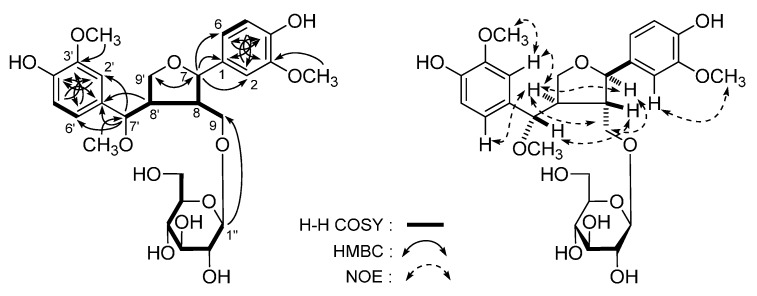
The main ^1^H-^1^H COSY, HMBC, and NOE correlations of **1**.

*Lippianoside A* (**1**), ${\left[\alpha \right]}_{D}^{25}$ +7.9° (in MeOH), white powder. Its molecular formula, C_27_H_36_O_12_, was determined from the molecular ion peak at *m*/*z* 575.2113 [M + Na]^+^ by HR-Q-TOF-ESI-MS measurement. Acid hydrolysis of **1** with 1 M HCl yielded d-glucose, which was identified on the basis of retention time (HPLC) and optical rotation [37,38]. ^1^H- and ^13^C-NMR (DMSO-*d*_6_, Table 1) spectra suggested the following moieties presented in **1**: a β-d-glucopyranosyl (δ 3.98 (1H, d, *J* = 8.0 Hz, H-1′′)), two ABX-type aromatic rings (δ 6.64 (1H, dd, *J* = 2.0, 8.0 Hz, H-6′), 6.71 (2H, *J* = 8.0 Hz, H-5 and 5′), 6.76 (1H, d, *J* = 2.0 Hz, H-2′), 6.77 (1H, dd, *J* = 2.0, 8.0 Hz, H-6), 6.88 (1H, d, *J* = 2.0 Hz, H-2)), three methoxy groups (δ 3.07, 3.73, 3.76 (3H each, all s, 7′, 3′, 3-OCH_3_)). In addition, the ^1^H and ^13^C-NMR spectra exhibited signals attributable to two methylenes bearing oxygen (δ 3.11 (1H, dd, *J* = 9.0, 9.0 Hz, H-9′a), 3.46 (1H, dd, *J* = 4.0, 9.0 Hz, H-9′b), 3.75 (1H, m, overlapped, H-9a), 4.05 (1H, dd, *J* = 4.5, 9.0 Hz, H-9b), two methines bearing oxygen (δ 3.97 (1H, d, *J* = 7.5 Hz, H-7′), 4.56 (1H, d, *J* = 7.5 Hz, H-7)), along with two aliphatic methines at δ 1.75 (1H, m, H-8) and 2.40 (1H, m, H-8′), respectively. According to the long-range correlations (Figure 3) observed from HMBC spectrum, the planar structure of **1** was determined. The coupling constant of H-7′ (*J* = 7.5 Hz) in **1** suggested an antiperiplanar orientation of H-7′ and H-8′. On the other hand, the CD spectrum of **1** (Δε: −121.7 (201 nm), −5.9 (228 nm)) was very similar to that of (7*R*,8*S*,7′*S*,8′*R*)-4,9,4′,7′-tetrahydroxy-3,3′-dimethoxy-7,9′-epoxylignan 9-*O*-β-d-glucopyranoside (Δε: −12.9 (205 nm), −2.5 (234 nm)) [39], which indicated the absolute configuration of **1** was 7*R*,8*S*,7′*S*,8′*R*. Furthermore, the NOE correlations between δ_H_ 4.56 (H-7) and δ_H_ 2.40 (H-8′) and 3.75 (H-9a); δ_H_ 1.75 (H-8) and δ_H_ 3.97 (H-7′); and δ_H_ 2.40 (H-8′) and δ_H_ 3.75 (H-9a), 6.64 (H-6′) and 6.76 (H-2′) observed in the NOESY spectrum confirmed the accuracy of configuration analysis. Finally, the structure of **1** was determined as (7*R*,8*S*,7′*S*,8′*R*)-4,9,4′-trihydroxy-3,3′,7′-trimethoxy-7,9′-epoxylignan 9-*O*-β-d-glucopyranoside.

**Table 1:** The ^1^H- and ^13^C-NMR data of **1**.

No.	1 ^a^		1 ^b^	
	δ_C_	δ_H_ (*J* in Hz)	δ_C_	δ_H_ (*J* in Hz)
1	133.2	-	134.8	-
2	110.4	6.88 (d, 2.0)	111.6	6.97 (d, 1.5)
3	147.2	-	149.0	-
4	145.5	-	147.2	-
5	115.0	6.71 (d, 8.0)	116.1	6.77 (d, 8.0)
6	118.5	6.77 (dd, 2.0, 8.0)	120.3	6.86 (dd, 1.5, 8.0)
7	82.7	4.56 (d, 7.5)	85.3	4.76 (d, 7.0)
8	49.7	1.75 (m)	51.6	1.96 (m)
9	69.5	3.75 (m, overlapped)	70.4	3.17 (dd, 4.0, 10.0)
		4.05 (dd, 4.5, 9.0)		3.55 (dd, 6.5, 10.0)
3-OCH_3_	55.5	3.76 (s)	56.7	3.86 (s)
1′	130.7	-	132.7	-
2′	110.9	6.76 (d, 2.0)	112.1	6.79 (d, 1.5)
3′	147.5	-	149.3	-
4′	145.9	-	147.6	-
5′	115.0	6.71 (d, 8.0)	116.2	6.76 (d, 8.5)
6′	120.2	6.64 (dd, 2.0, 8.0)	121.8	6.70 (dd, 1.5, 8.0)
7′	84.5	3.97 (d, 7.5)	87.1	4.01 (d, 9.0)
8′	48.5	2.40 (m)	50.3	2.52 (m)
9′	68.7	3.11 (dd, 9.0, 9.0)	71.8	3.97 (dd, 9.0, 9.0)
		3.46 (dd, 4.0, 9.0)		4.18 (dd, 4.0, 9.0)
3′-OCH_3_	55.7	3.73 (s)	56.6	3.82 (s)
7′-OCH_3_	55.4	3.07 (s)	56.7	3.16 (s)
1′′	102.8	3.98 (d, 7.5)	104.5	4.02 (d, 8.0)
2′′	73.4	2.91 (dd, 7.5, 9.0)	75.2	3.11 (dd, 8.0, 9.0)
3′′	76.6	3.11 (dd, 9.0, 9.0)	78.2	3.29 (dd, 9.0, 9.0)
4′′	69.8	3.03 (dd, 9.0, 9.0)	71.7	3.28 (dd, 9.0, 9.0)
5′′	76.7	3.01 (m)	77.9	3.16 (m)
6′′	60.9	3.45 (dd, 5.5, 12.5)	62.8	3.66 (dd, 5.5, 12.0)
		3.61 (dd, 2.5, 12.5)		3.82 (dd, 2.5, 12.0)

^a^: determined in DMSO-*d*_6_; ^b^*:* determined in CD_3_OD.

**Figure 4:**
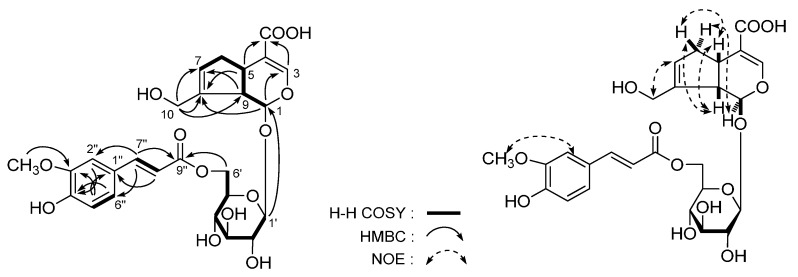
The main ^1^H-^1^H COSY, HMBC and NOE correlations of **2**.

*Lippianoside B* (**2**) was obtained with negative optical rotation (${\left[\alpha \right]}_{D}^{25}$ −8.9° in MeOH). The molecular formula of **2** was revealed as C_26_H_30_O_13_ by HR-Q-TOF-ESI-MS (*m/z* 549.1604 [M − H], calcd for C_26_H_29_O_13_, 549.1614). Treatment of **2** with 1 M HCl gave d-glucose, which was identified by HPLC analysis [37,38]. ^1^H-, ^13^C-NMR (CD_3_OD, Table 2) and various 2D NMR spectra (Figure 4), indicated the presence of an iridoid moiety, a *trans*-feruloyl, and a β-d-glucopyranosyl in **2**. In the HMBC experiment (Figure 4), the long-range correlations between the following proton and carbon pairs were observed: δ_H_ 4.98 (H-1) and δ_C_ 145.0 (C-8), 152.9 (C-3); δ_H_ 3.13 (H-5) and δ_C_ 145.0 (C-8), 171.0 (C-11); δ_H_ 2.69 (H-9) and *δ*_C_ 113.3 (C-4), 128.9 (C-7); δ_H_ 4.19, 4.25 (H_2_-10) and δ_C_ 46.7 (C-9), 128.9 (C-7), 145.0 (C-8); δ_H_ 4.73 (H-1′) and δ_C_ 98.9 (C-1); δ_H_ 4.40, 4.44 (H_2_-6′) and δ_C_ 169.1 (C-9′′). Finally, in the NOESY spectrum, the NOE correlations between δ_H_ 4.98 (H-1) and δ_H_ 1.96 (Hα-6); δ_H_ 3.13 (H-5) and δ_H_ 2.69 (H-9) and 2.75 (Hβ-6); and δ_H_ 2.75 (Hβ-6) and δ_H_ 2.69 (H-9) suggested the relative configuration of **2** was 1α,5β,9β. On the other hand, the ^1^H and ^13^C-NMR spectra of **2** were superimposable on those of 6′-*O*-*trans*-*p*-coumaroyl geniposidic acid [40], except for the signals due to *trans*-feruloyl group at 6′-position. Consequently, the structure of lippianoside B was elucidated to be 6′-*O*-*trans*-feruloyl geniposidic acid (**2**).

**Table 2:** The ^1^H- and ^13^C-NMR data of **2** in CD_3_OD.

No.	δ*_C_*	δ_H_ (*J* in Hz)	No.	δ_C_	δ_H_ (*J* in Hz)
1	98.9	4.98 (d, 8.0)	3′	77.8	3.43 (dd, 8.5, 9.0)
3	152.9	7.49 (s)	4′	71.9	3.37 (dd, 9.0, 9.0)
4	113.3	-	5′	75.7	3.56 (m)
5	37.1	3.13 (q like, 8.0, 8.0)	6′	64.4	4.40 (dd, 6.0, 12.0)
6	39.9	1.96 (br. dd, *ca*. 8, 16)			4.44 (dd, 2.5, 12.0)
		2.75 (br. dd, *ca*. 8, 16)	1′′	127.7	-
7	128.9	5.76 (br. s)	2′′	111.6	7.17 (br. s)
8	145.0	-	3′′	149.4	-
9	46.7	2.69 (dd, 8.0, 8.0)	4′′	150.7	-
10	61.6	4.19 (d, 14.0)	5′′	116.5	6.81 (d, 8.0)
		4.25 (d, 14.0)	6′′	124.3	7.05 (br. d, *ca*. 8)
11	171.0	-	7′′	147.1	7.59 (d, 16.0)
1′	100.7	4.73 (d, 7.5)	8′′	115.3	6.35 (d, 16.0)
2′	74.8	3.27 (dd, 7.5, 8.5)	9′′	169.1	-
			3′′-OCH_3_	56.5	3.88 (s)

**Figure 5:**
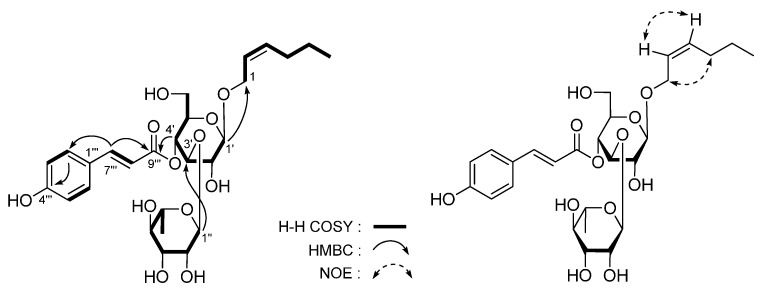
The main ^1^H-^1^H COSY, HMBC and NOE correlations of **3**.

*Lippianoside C* (**3**), ${\left[\alpha \right]}_{D}^{25}$ −61.4° (in MeOH), was isolated as a white powder. Its molecular formula, C_27_H_38_O_12_, was established by HR-Q-TOF-ESI-MS with *m*/*z* 557.2267 [M + Na]^+^ (calcd for C_27_H_38_O_12_Na, 557.2255). Its IR spectrum showed absorption bands due to hydroxyl (3384 cm^−1^), α,β-unsaturated carbonyl (1701 cm^−1^), aromatic ring (1603, 1514 cm^−1^), and ether function (1064 cm^−1^). Acid hydrolysis of **3** with 1 M HCl gave d-glucose and L-rhamnose [37,38]. The ^1^H-, ^13^C-NMR (CD_3_OD, Table 3) and various kinds of 2D NMR (Figure 5) including ^1^H-^1^H COSY, HMQC, and HMBC showed signals assignable to 2-hexene-1-alcohol, *trans*-*p*-coumaroyl, β-d-glucopyranosyl, and α-l-rhamnopyranosyl. In the HMBC experiment, the long-range correlations were observed between δ_H_ 4.36 (H-1′) and δ_C_ 70.7 (C-1); δ_H_ 5.19 (H-1′′) and δ_C_ 81.7 (C-3′); and δ_H_ 4.92 (H-4′) and δ_C_ 168.4 (C-9′′′), and the connectivities of the above-mentioned moieties were elucidated. Finally, in the NOESY experiment, the NOE correlations observed between δ_H_ 2.39 (H_2_-4) and δ_H_ 3.56, 3.87 (H_2_-1) as well as δ_H_ 5.47 (H-2) and δ_H_ 5.38 (H-3) indicated the double bond configuration of 2-position in **3** was *Z*.

**Figure 6:**
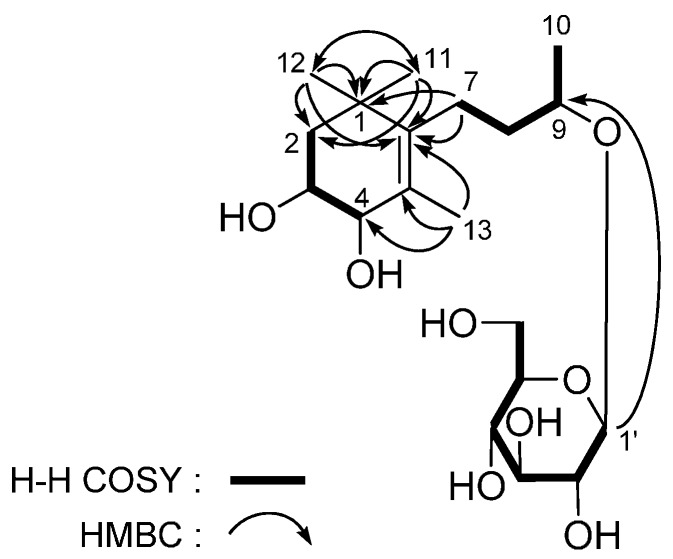
The main ^1^H-^1^H COSY and HMBC correlations of **4**.

*Lippianoside D* (**4**) was a white powder with negative optical rotation (${\left[\alpha \right]}_{D}^{25}$ −62.1° in MeOH). Its elemental composition was determined to be C_19_H_34_O_8_ by HR-Q-TOF-ESI-MS observed at *m/z* 413.2158 [M + Na]^+^. The ^1^H and ^13^C-NMR (CD_3_OD, Table 4) showed signals due to four methyl (δ 1.05, 1.07, 1.78 (3H each, all s, H_3_-11, 12, 13), 1.20 (3H, d, *J* = 6.0 Hz, H_3_-10)), three methylene (δ (1.38 (1H, dd, *J* = 5.0, 13.0 Hz), 1.74 (1H, ddd, *J* = 13.0, 13.0 Hz), H_2_-2), 1.57, 1.66 (1H each, both m, H_2_-8), (1.99 (1H, ddd, *J* = 5.0, 13.0, 13.0 Hz), 2.27 (1H, ddd, *J* = 5.0, 13.0, 13.0 Hz), H_2_-7)), three methine bearing an oxygen function (δ 3.71 (1H, ddd, *J* = 4.0, 5.0, 13.0 Hz, H-3), 3.73 (1H, br. d, *ca*. *J* = 4 Hz, H-4), 3.90 (1H, m, H-9)), together with a β-d-glucopyranosyl (δ 4.35 (1H, d, *J* = 7.5 Hz, H-1′)). The ^1^H-^1^H COSY experiment indicated the presence of partial structure written in bold lines (Figure 6). According to the long-range correlations (Figure 6) observed from HMBC spectrum, the planar structure of **4** was determined. Finally, treatment of **4** with 1 M HCl liberated d-glucose [37,38]. Enzymatic hydrolysis of it with β-glucosidase gave (3*S*,4*R*,9*R*)-3,4,9-trihydroxymegastigman-5-ene [27] as aglycon. Then, the structure lippianoside D was determined to be (3*S*,4*R*,9*R*)-3,4,6-trihydroxymegastigman-5-ene 9-*O*-β-d-glucopyranoside (**4**).

**Table 3:** The ^1^H- and ^13^C-NMR data of **3** in CD_3_OD.

No.	δ*_C_*	δ_H_ (*J* in Hz)	No.	δ_C_	δ_H_ (*J* in Hz)
1	70.7	3.56 (m) 3.87 (m)	2′′	72.4	3.91 (dd, 1.0, 3.0)
2	134.6	5.47 (m)	3′′	72.1	3.57 (dd, 3.0, 9.5)
3	125.9	5.38 (m)	4′′	73.8	3.27 (dd, 9.5, 9.5)
4	28.8	2.39 (q like, *ca*. 7)	5′′	70.4	3.58 (m)
5	21.6	2.08 (m)	6′′	18.5	1.08 (d, 6.5)
6	14.7	0.97 (t, 7.5)	1′′′	127.1	-
1′	104.2	4.36 (d, 8.0)	2′′′	131.4	7.46 (d, 8.5)
2′	76.2	3.38 (dd, 8.0, 8.5)	3′′′	117.0	6.80 (d, 8.5)
3′	81.7	3.82 (dd, 8.5, 9.0)	4′′′	161.6	-
4′	70.7	4.92 (dd, 9.0, 9.0)	5′′′	117.0	6.80 (d, 8.5)
5′	76.1	3.54 (m)	6′′′	131.4	7.46 (d, 8.5)
6′	62.4	3.52 (dd, 6.0, 12.0)	7′′′	147.7	7.66 (d, 16.0)
		3.62 (br. d, *ca*. 12)	8′′′	114.8	6.34 (d, 16.0)
1′′	103.1	5.19 (d, 1.0)	9′′′	168.4	-

**Table 4:** The ^1^H- and ^13^C-NMR data of **4**.

No.	4 ^a^		4 ^b^	
	δ*_C_*	δ_H_ (*J* in Hz)	δ_C_	δ_H_ (*J* in Hz)
1	38.8	-	37.9	-
2	42.0	1.38 (dd, 5.0, 13.0)	42.4	1.72 (dd, 3.0, 12.5)
		1.74 (dd, 13.0, 13.0)		2.20 (dd, 12.5, 12.5)
3	68.1	3.71 (ddd, 4.0, 5.0, 13.0)	67.1	4.12 (ddd, 4.0, 5.5, 12.5)
4	73.1	3.73 (br. d, *ca*. 4)	72.3	4.19 (br. d, *ca*. 4)
5	128.1	-	128.4	-
6	143.7	-	141.6	-
7	25.6	1.99 (ddd, 5.0, 13.0, 13.0)	25.0	2.11 (ddd, 4.5, 13.0, 13.0)
		2.27 (ddd, 5.0, 13.0, 13.0)		2.48 (ddd, 4.5, 13.0, 13.0)
8	38.3	1.57 (m), 1.66 (m)	37.9	1.65 (m), 1.85 (m)
9	76.1	3.90 (m)	74.8	4.14 (m)
10	19.8	1.20 (d, 6.0)	19.9	1.31 (d, 6.5)
11	27.7	1.05 (s)	27.4	1.03 (s)
12	30.0	1.07 (s)	29.6	1.04 (s)
13	18.5	1.78 (s)	18.5	1.97 (s)
1′	102.3	4.35 (d, 7.5)	102.4	4.93 (d, 7.5)
2′	75.2	3.16 (dd, 7.5, 8.5)	75.2	4.04 (dd, 7.5, 8.5)
3′	78.2	3.36 (dd, 8.5, 9.0)	78.6	4.29 (dd, 8.5, 9.0)
4′	71.9	3.30 (dd, 9.0, 9.0)	71.9	4.23 (dd, 9.0, 9.0)
5′	77.9	3.26 (m)	78.3	3.97 (m)
6′	63.0	3.67 (dd, 5.5, 12.0)	63.0	4.36 (dd, 5.0, 11.5)
		3.85 (dd, 2.0, 12.0)		4.55 (dd, 2.0, 11.5)

^a^ measured in CD_3_OD; ^b^ measured in C_5_D_5_N.

**Figure 7:**
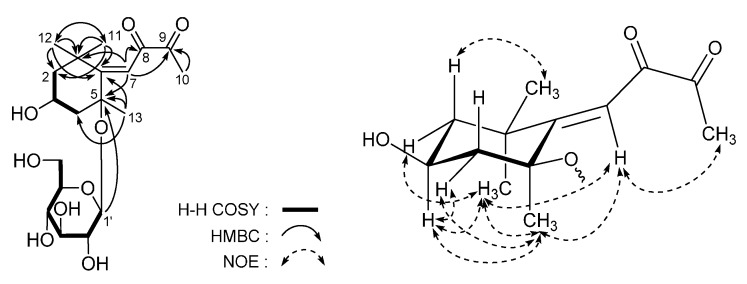
The main ^1^H-^1^H COSY, HMBC, and NOE correlations of **5**.

*Lippianoside E* (**5**) was obtained as a white powder. Its molecular formula, C_19_H_30_O_9_, was determined from the positive HR-Q-TOF-ESI-MS. The ^1^H- and ^13^C-NMR (CD_3_OD, Table 5) spectra showed signals assignable to four methyls (δ 1.15, 1.37, 1.47, 2.19 (3H each, all s, H_3_-12, 11, 13, 10)), two methylenes (δ (1.33 (1H, dd, *J* = 12.0, 12.0 Hz), 1.92 (1H, ddd, *J* = 2.5, 5.0, 12.0 Hz), H_2_-2), (1.37 (1H, dd, *J* = 12.0, 12.0 Hz), 2.48 (1H, ddd, *J* = 2.5, 4.5, 12.0 Hz), H_2_-4)), a methine bearing an oxygen function (δ 4.32 (1H, m, H-3)), a three-substituted double bond (δ 5.89 (1H, s, H-7)), two carboxyl groups (δ_C_ 200.7 (C-9), 213.0 (C-8)), and a β-d-glucopyranosyl (δ 4.52 (1H, d, *J* = 7.5 Hz, H-1′)). Selected long-range correlations observed in the HMBC experiment were shown in Figure 7. Treatment of **5** with 1 M HCl yielded d-glucose [37,38]. In the NOESY experiment, NOE correlations were observed between the following proton pairs: δ_H_ 1.92 (H*eq*-2) and δ_H_ 1.37 (H_3_-11) and 4.32 (H-3); δ_H_ 4.32 (H-3) and δ_H_ 1.37 (H_3_-11) and 1.47 (H_3_-13); and δ_H_ 5.89 (H-7) and δ_H_ 1.37 (H_3_-11), 1.47 (H_3_-13), and 2.19 (H_3_-10), which suggested the configuration of **5** was 3S*5S*6*E*.

**Table 5:** The ^1^H- and ^13^C-NMR data of **5**.

No.	5 ^a^		5 ^b^	
	δ*_C_*	δ_H_ (*J* in Hz)	δ_C_	δ_H_ (*J* in Hz)
1	37.1	-	35.4	-
2	49.9	1.33 (dd, 12.0, 12.0)	49.1	1.21 (dd, 12.5, 12.5)
		1.92 (ddd, 2.5, 5.0, 12.0)		1.79 (ddd, 3.0, 4.5, 12.0)
3	63.8	4.32 (m)	61.0	4.15 (m)
4	48.1	1.37 (dd, 12.0, 12.0)	45.9	1.19 (dd, 12.0, 12.0)
		2.48 (ddd, 2.5, 4.5, 12.0)		2.38 (ddd, 3.0, 5.0, 12.0)
5	78.7	-	76.9	-
6	141.6	-	117.4	-
6	119.1	-	117.4	-
7	101.4	5.89 (s)	99.8	5.86 (s)
8	213.0	-	210.6	-
9	200.7	-	197.5	-
10	26.7	2.19 (s)	26.2	2.12 (s)
11	30.1	1.37 (s)	29.0	1.29 (s)
12	32.5	1.15 (s)	31.7	1.05 (s)
13	26.6	1.47 (s)	26.3	1.33 (s)
1′	98.7	4.52 (d, 7.5)	96.8	4.36 (d, 7.0)
2′	75.3	3.14 (dd, 7.5, 9.0)	73.6	2.91 (dd, 7.0, 8.0)
3′	78.6	3.35 (dd, 9.0, 9.0)	77.3	3.16 (dd, 8.0, 9.0)
4′	71.7	3.25 (dd, 9.0, 9.0)	70.1	3.01 (dd, 9.0, 9.0)
5′	77.8	3.22 (m)	76.6	3.06 (m)
6′	62.9	3.61 (dd, 5.5, 12.0)	61.1	3.37 (dd, 6.0, 12.0)
		3.81 (dd, 2.0, 12.0)		3.62 (dd, 2.0, 12.0)

^a^ measured in CD_3_OD; ^b^ measured in DMSO-*d*_6_.

**Figure 8:**
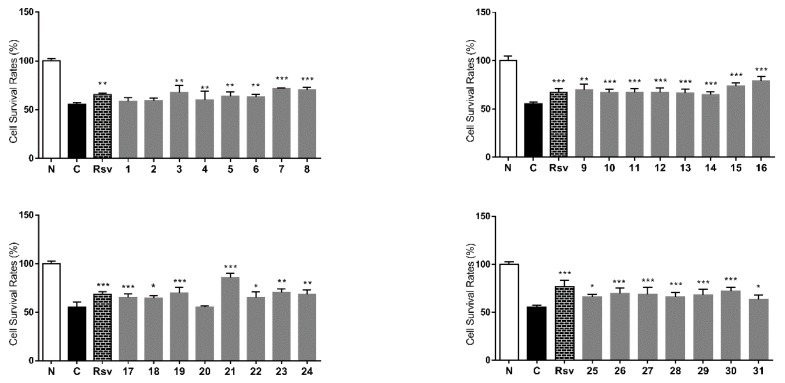
Cell survival rate of **1**–**31** on L6 cells treated with antimycin A. Values represent the mean ± SD of determinations (*n* = 8). * *p* < 0.05; ** *p* < 0.01; *** *p* < 0.001 *vs.* control group. Final administrated concentration of revestrol was 10 μM, and **1**–**31** was 30 μM.

Antimycin A, an electron transport chain inhibitor in mitochondria between cytochromes b and c, can produce ROS in cells, causing the leakage of superoxide radicals from cell mitochondria by inhibiting mitochondrial electron transport [41]. Compared with normal group, 20 µg/mL antimycin A induced significant L6 cell injury at a rate of 50%, while 10 µM resveratrol showed increased cell survival rate effects compared with the antimycin treated group. Except for **1**, **2** and **20**, all the compounds isolated from *L. triphylla* displayed significant protective effects against antimycin A-induced L6 cell injury at 30 µM, and **21** showed strongest protective activity (Figure 8).

**Figure 9:**
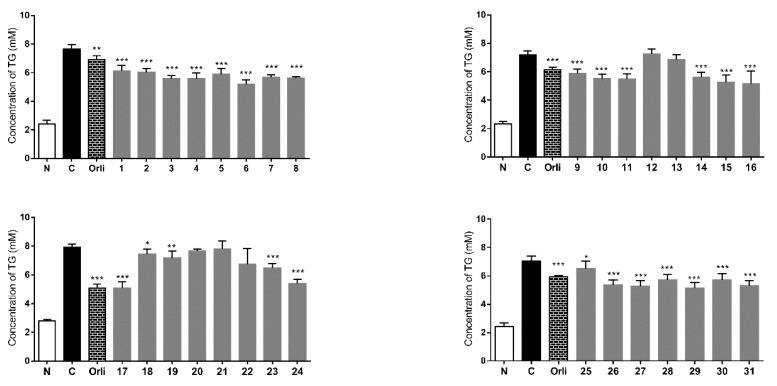
TG accumulation inhibitory effects of **1**–**31** in HepG2 cells. TG relative concentration: percentage of control group, which set as 100%. Values represent the mean ± SD of determinations (*n* = 8). * *p* < 0.05; ** *p* < 0.01; *** *p* < 0.001 *vs.* control group. Final administrated concentration of **1**–**31** was 10 μM, and oristat was 0.5 μM.

Intracellular excess lipid accumulation (especially in liver and muscle) is a mediator of metabolic syndrome, which is comprised of a cluster of risk factors such as diabetes, hyperlipidemia, and hypertension. Free fatty acid (FFA) induced TG accumulation in HepG2 cells is commonly used for research on lipid metabolism regulation effects [42]. As shown in Figure 9, intracellular lipid contents were significantly increased after 0.2 mM oleic acid treatment. This accumulation effects were inhibited by orlistat at 0.5 µM. The TG accumulation inhibitory effects of the isolates were tested. Except for compounds **12**, **13** and **20**–**22**, all isolates displayed inhibitory effects on TG accumulation in FFA induced HepG2 cells.

## 3. Experimental Section

### 3.1. General

The following instruments were used to obtain physical data: Optical rotations were determined on a Rudolph Autopol IV automatic polarimeter (Rudolph Research Analytical, Hackettstown, NJ, USA, l = 50 mm), IR and UV spectra were recorded on Varian 640-IR FT-IR (Varian Australia Pty Ltd., Mulgrave, Australia)and Varian Cary 50 UV-VIS spectrophotometer (Varian, Inc., Hubbardsdon, MA, USA), respectively. ^1^H- and ^13^C-NMR spectra were measured on a Bruker 500 MHz NMR spectrometer (Bruker BioSpin AG Industriestrasse 26 CH-8117, Fällanden, Switzerland) at 500 MHz for ^1^H- and 125 MHz for ^13^C-NMR, with tetramethylsilane (TMS) as an internal standard. Positive- and negative-ion HR-ESI-Q-TOF-MS were recorded on an Aglient 6520 Q-TOF mass spectrometer (Agilent Corp., Santa Clara, CA, USA).

The following experimental conditions were used for chromatography: A macroporous synthetic resin (D101) (Haiguang Chemical Co., Ltd., Tianjin, China), Silica gel (74–149 μm, Qingdao Haiyang Chemical Co., Ltd., Qingdao, China), and ODS (50 μm, YMC Co., Ltd., Tokyo, Japan). HPLC was performed on ODS (Cosmosil 5C18-MS-II, Tokyo, Japan; Φ = 20 mm, L = 250 mm, flow rate 9.0 mL/min), and the eluate was monitored with a UV detector (Shimadzu RID-10ª UV-vis, Shimadzu Co. Ltd., Kyoto, Japan).

### 3.2. Plant Material

The aerial parts of *Lippia triphylla* were collected in Rwanda, Africa, and identified by Dr. Tianxiang Li at Tianjin University of TCM as M. indica L. A voucher specimen was deposited at the Academy of Traditional Chinese Medicine of Tianjin University of TCM.

### 3.3. Extraction and Isolation

The dried aerial parts of *L. triphylla* (2.85 kg) were extracted with 95% EtOH under reflux. Evaporation of the solvent under reduced pressure to yield a 95% ethanol–water extract (403 g). The extract partitioned with CHCl_3_–H_2_O (1:1, *v*/*v*) to give CHCl_3_ (105 g) and H_2_O (258 g) layers. The H_2_O layer (215 g) was subjected to D101 macroporous resin CC and eluted with H_2_O and 95% EtOH, successively, to yield H_2_O (131 g) and 95% EtOH (63 g) eluates, respectively.

The 95% EtOH eluate (50.0 g) was separated by SiO_2_ gel CC (CHCl_3_ → CHCl_3_-MeOH (100:2 → 100:4 → 100:6, *v*/*v*) → CHCl_3_–MeOH–H_2_O (10:3:1 → 7:3:1 → 6:4:1, *v*/*v*/*v*, lower layer) → MeOH) to afford 16 fractions (Fr. 1–16). Fraction 3 (1.0 g) was isolated by SiO_2_ gel and Sephadex LH-20 CC to give nepetin (**16**, 12.0 mg). Fraction 6 (1.2 g) was subjected to ODS, Sephadex LH-20 CC, and purified by PHPLC to yield benzyl alcohol *O*-β-d-glucopyranoside (**30**, 5.2 mg) and icariside H_1_ (**31**, 4.3 mg). Fraction 7 (7.3 g) was separated by PHPLC and Sephadex LH-20 CC with different analysis conditions to afford lippianosides B (**2**, 19.0 mg) and C (**3**, 5.0 mg), martynoside (**10**, 7.0 mg), isomartynoside (**11**, 7.0 mg), nepitrin (**17**, 12.0 mg), dehydrodiconiferyl glucosides D (**18**, 20.0 mg) and E (**19**, 13.0 mg), (+)-lariciresinol-9-*O*-β-d-glucopyranoside (**20**, 28.0 mg), (+)-pinoresinol 4-*O*-β-d-glucoside (**21**, 6.0 mg), and dihydrovomifoliol-*O*-β-d-glucopyranoside (**22**, 40.0 mg). Fraction 8 (5.8 g) was purified by PHPLC, and as a result, lippianosides A (**1**, 14.0 mg), D (**4**, 10.1 mg), and E (**5**, 11.0 mg), jionoside C (**6**, 8.4 mg), and turpinionoside D (**23**, 7.2 mg) were given. Fraction 11 (8.6 g) was separated by PHPLC and ODS CC to yield *trans*-acteoside (**7**, 17.3 mg), isoverbascoside (**8**, 55.1 mg), *cis*-acteoside (**9**, 36.1 mg), β-hydroxyacteoside (**12**, 8.3 mg), campneoside I (**13**, 60.6 mg), and cistanoside F (**14**, 70.4 mg).

The above-mentioned CHCl_3_ layer (85.0 g) was subjected to Silica gel CC (CHCl_3_ → CHCl_3_–MeOH (100:1 → 100:3 → 100:5 → 100:7, *v*/*v*) → MeOH) to give 13 fractions (Fr. 1′–13′). Fraction 5 (29.3 g) was purified by Sephadex LH-20 and Silica gel CC, along with PHPLC to afford jaceosidin (**15**, 11.2 mg), 9-hydroxymegastigm-5-en-4-one (**24**, 2.4 mg), (−)-loliolide (**25**, 13.1 mg), (6*S*)-3,7-dimethyl-7-hydroxy-2(*Z*)-octen-6-olide (**27**, 11.5 mg), ursolic acid (**28**, 20.9 mg), and avicennone A (**29**, 5.4 mg).

*Lippianoside A* (**1**): White powder; ${\left[\alpha \right]}_{D}^{25}$ +7.9° (*c* = 0.61, MeOH); CD (*c* = 0.00166 M, MeOH) Δε (λ nm) −121.7 (201), −5.9 (228); UV (MeOH) λ_max_ (log ε) 203 (4.89), 229 (4.07), 279 (3.70); IR (KBr) ν_max_ 3384, 2930, 1603, 1514, 1451, 1263, 1163, 1064, 1039, 833 cm^−1^; ^1^H- and ^13^C-NMR (CD_3_OD) data see Table 1; Positive-ion mode HR-Q-TOF-ESI-MS *m*/*z* 575.2113 (calcd for C_27_H_36_O_12_Na [M + Na]^+^, 575.2099).

*Lippianoside B* (**2**): White powder; ${\left[\alpha \right]}_{D}^{25}$ −8.9° (*c* = 0.79, MeOH); UV (MeOH) λ_max_ (log ε) 218 (4.21), 231 (4.20), 286 (3.92, sh), 323 (4.09); IR (KBr) ν_max_ 3552, 2940, 2840, 1690, 1596, 1517, 1448, 1161, 1025, 944, 897 cm^−1^. ^1^H- and ^13^C-NMR (CD_3_OD) data see Table 2. Negative-ion mode HR-Q-TOF-ESI-MS *m*/*z* 549.1604 [M − H]^−^ (calcd for C_26_H_29_O_13_, 549.1614).

*Lippianoside C* (**3**): White powder; ${\left[\alpha \right]}_{D}^{25}$ −61.4° (*c* = 0.37, MeOH); UV (MeOH) λ_max_ (log ε) 312 (4.21); IR (KBr) ν_max_ 3384, 2930, 1701, 1603, 1514, 1263, 1163, 1064, 1039, 833 cm^−1^; ^1^H- and ^13^C-NMR (CD_3_OD) data see Table 3. Positive-ion mode HR-Q-TOF-ESI-MS *m*/*z* 557.2267 (calcd for C_27_H_38_O_12_Na [M + Na]^+^, 557.2255).

*Lippianoside D* (**4**): White powder; ${\left[\alpha \right]}_{D}^{25}$ −62.1° (*c* = 0.86, MeOH); IR (KBr) ν_max_ 3360, 2964, 2929, 1621, 1377, 1262, 1073, 1026, 875 cm^−1^; ^1^H- and ^13^C-NMR (CD_3_OD) data see Table 4; Positive-ion mode HR-Q-TOF-ESI-MS *m*/*z* 413.2158 (calcd for C_19_H_34_O_8_Na [M + Na]^+^, 413.2146).

*Lippianoside E* (**5**): White powder; ${\left[\alpha \right]}_{D}^{25}$ −87.0° (*c* = 0.20, MeOH); UV (MeOH) λ_max_ (log ε) 229 (3.62); IR (KBr) ν_max_ 3350, 2915, 2848, 1665, 1405, 1248, 1070, 1070, 1036 cm^−1^; ^1^H- and ^13^C-NMR (DMSO-*d*_6_) data see Table 5; Positive-ion mode HR-Q-TOF-ESI-MS *m*/*z* 425.1720 (calcd for C_19_H_30_O_9_Na [M + Na]^+^, 425.1782).

Acid Hydrolysis of **1**–**5***:* A solution of compounds **1**–**5** (each 2.0 mg) in 1 M HCl (1.0 mL) was heated under reflux for 3 h. After cooling, the reaction mixture was extracted with EtOAc. The aqueous layer was subjected to HPLC analysis under the following conditions, respectively: HPLC column, Kaseisorb LC NH_2_-60-5, 4.6 mm i.d. 250 mm (Tokyo Kasei Co., Ltd., Tokyo, Japan); detection, optical rotation (Shodex OR-2 (Showa Denko Co., Ltd., Tokyo, Japan); mobile phase, CH_3_CN–H_2_O (75:25, *v*/*v*); flow rate 0.7 mL/min). Identification of l-rhamnose (i) from **3**; and D-glucose (ii) from **1**–**5** present in the aqueous layer was carried out by comparison of its retention time and optical rotation with those of authentic sample, *t*_R_: (i) 7.5 min (l-rhamnose, negative optical rotation); and (ii) 14.1 min (d-glucose, positive optical rotation).

Enzymatic hydrolysis of **4** with β-glucosidase*:* A solution of **4** (7.0 mg) in H_2_O (2.5 mL) was treated with β-glucosidase (5.0 mg, Almond, Sigma-Aldrich, Co., St. Louis, MO, USA), and the solution was stirred at 37 °C for 20 h. After cooling, the reaction mixture was extracted with EtOAc. The EtOAc solvent was removed under reduced pressure to give (3*S*,4*R*,9*R*)-3,4,9-trihydroxymegastigman-5-ene.

Mitochondrial oxidative stress protective effects assay: Antimycin A was used to induce mitochondrial oxidative stress. Briefly, L6 cells (Cell Resource Center, IBMS, CAMS/PUMC, Beijing, China) were plated at a density of 5 × 10^4^ cells/well in Dulbecco’s modified Eagle’s medium (DMEM, Thermo Scientific, Logan, UT, USA) supplemented with 10% calf serum (Thermo Scientific) in a 96-well plate and were incubated at 37 °C for 24 h. Cells were treated with or without 30 μM sample DMSO solution (final DMSO concentration was 0.5%). One hour later, medium was removed and 20 μg/mL antimycin A (Sigma Co. Ltd.) in 200 μL DMEM was added to each well. The MTT assay was performed 24 h later to detect the cell survival rate. Resveratrol was used as positive control.

TG accumulation inhibitory effects assay: The hepatic cell line HepG2 (IBMS, CAMS/PUMC, Beijing, China) was maintained in high glucose Minimum Essential Medium (MEM) supplemented with 10% fetal bovine serum (FBS) and 1% penicillin–streptomycin under a humidified atmosphere of 5% CO_2_ in air. After growth to 80% confluence, cells were seeded at 4 × 10^4^ cells/mL on a 48-well dish. After 24 h incubation, the medium was switched to high glucose MEM and supplemented with 10% FBS and 0.2 mM oleic acid sodium salt, together with sample DMSO solution (final concentration of DMSO was less than 0.1%). After 48 h incubation, the amount of intracellular triglycerides was determined with a Triglycerides kit (BioSino Bio-technology and Science Inc., Beijing, China) after cell lysis. Orlistat was used as positive control.

Statistical analysis: Values are expressed as mean ± SD. Analyses on the grouped data were performed using SPSS 11.0. Significant differences between means were evaluated by one-way analysis of variance (ANOVA) and Tukey’s Studentized range test was used for *post hoc* evaluations. A *p* value of <0.05 was considered to indicate statistical significance.

## 4. Conclusions

In summary, five new, along with 26 known, compounds were identified from the 95% EtOH extract of *L. triphylla* aerial parts collected from Rwanda, Africa. Their structures were elucidated by chemical and spectroscopic methods. Among the known compounds, 11, and 17–30 were isolated from *Lippia* genus for the first time. In addition, 12, 13, and 16 were obtained from this species for the first time. All compounds were tested for their antioxidant and triglyceride accumulation inhibition effects in L6 cells and HepG2 cells, respectively. The results indicated that, except for 1, 2 and 20, all compounds isolated from *L. triphylla* displayed significant protective effects against antimycin A-induced L6 cell injury at 30 µM, and 21 showed the strongest protective activity. Meanwhile, 1–11, 14–19, and 23–26 displayed inhibitory effects on TG accumulation in FFA induced HepG2 cells. Our study provides partial scientific support for the development and utilization of *L. triphylla* aerial parts.
